# A Highly Troglomorphic New Genus of Sminthuridae (Collembola, Symphypleona) from the Brazilian Semiarid Region †

**DOI:** 10.3390/insects13070650

**Published:** 2022-07-19

**Authors:** Paolla Gabryelle Cavalcante de Souza, Gleyce da Silva Medeiros, Rodrigo Lopes Ferreira, Marconi Souza-Silva, Bruno Cavalcante Bellini

**Affiliations:** 1 Department of Botany and Zoology, Biosciences Center, Federal University of Rio Grande do Norte (UFRN), Highway BR-101, Lagoa Nova, Campus Universitario, Natal 59072-970, RN, Brazil; paollasouzac@gmail.com; 2 Centro de Estudos em Biologia Subterrânea, Departamento de Ecologia e Conservação, Instituto de Ciências Naturais, Universidade Federal de Lavras (UFLA), Lavras 37200-900, MG, Brazil; drops@ufla.br (R.L.F.); marconisilva@ufla.br (M.S.-S.)

**Keywords:** Neotropical Region, new species, Sminthurinae, Sminthuroidea, *Temeritas*-group

## Abstract

**Simple Summary:**

A new genus and species of cave springtail are herein described. The new species has a remarkable morphology, suggesting it evolved within caves, such as the appendages and chaetae elongation, loss of body pigments, and 5 + 5 eyes reduced in size. The new species may also be occasionally cannibalistic, as one of the analyzed females devoured another male from her species.

**Abstract:**

Here, we describe the highly troglomorphic *Troglobentosminthurus* gen. nov. from Água Clara cave system, Caatinga domain, Bahia, Brazil. *Troglobentosminthurus luridus* gen. nov. sp. nov. has remarkably long antennae, legs and furca, and lacks body pigments, except for small orange eye patches which also show a reduction in the number of eyes (5 + 5) and lens sizes. The overall morphology of the genus, with long and highly sub-segmented antennae, resembles other Sminthurinae of the *Temeritas*-group, especially *Temeritas* Richards and *Galeriella* Ćurčić and Lučić. However, it is unique, especially in the combination of the number of antennae IV subsegments and eyes, frontal head chaetotaxy and empodial complex morphology. Two type specimens have remnants of a mite and another specimen from the new species in their gut contents, supporting the species may be occasional predators and even cannibals. We also provide identification keys and comparative tables to the subfamilies of Sminthuridae and the *Temeritas*-group of genera.

## 1. Introduction

The knowledge on the Brazilian Symphypleona strongly increased during the past two decades. In 2003, there were only 40 species recorded in the entire country, while in 2022, there are 73 nominal species recognized in Brazil [1,2]. Similarly to the Poduromorpha and Entomobryomorpha, this remarkable rise in the taxonomic understanding of the Symphypleona was mainly due to the description of new species rather than the register of previously described taxa from other countries [2]. In this context, the Brazilian fauna of Symphypleona is unique, with 57 endemic species (about 78% of its total), as well as some genera only found in Brazil, like *Arborianna* Bretfeld, 2002 [3] (Katiannidae), *Keratosminthurus* Zeppelini 2020 in [4], and *Varelasminthurus* Silva, Palacios-Vargas and Bellini, 2015 [5] (both Sminthuridae).

The cave fauna of Brazilian springtails is mainly represented by species of *Acherontides* Bonet, 1945 [6] (Poduromorpha); *Cyphoderus* Nicolet, 1842 [7], *Troglobius* Palacios-Vargas and Wilson, 1990 [8], *Trogolaphysa* Mills, 1938 [9], *Pseudosinella* Schäffer, 1897 [10], *Coecobrya* Yosii, 1956 [11] (Entomobryomorpha); *Arrhopalites* Börner, 1906 [12], *Pararrhopalites* Bonet and Tellez, 1947 [13] and *Keratosminthurus* (Symphypleona), summing up more than 40 species [2,4,14,15,16,17,18,19,20,21]. Although many recent efforts aimed to describe the Brazilian cave fauna of Collembola, as in [4,15,16,18,19,21], it is noteworthy that most subterranean ecosystems in the country are undersampled for invertebrates in general (including the springtails fauna), especially in the semiarid landscapes. On the other hand, preliminary data supports that the Caatinga, a unique semiarid domain only found in Brazil, can host complex cave systems which shelter a high number of troglomorphic endemic invertebrates, many of them potentially troglobitic [22,23].

Sminthuridae Lubbock, 1862 is the largest and one of the most widely distributed families of Symphypleona [24]. Due to its species and genera richness, different attempts were made to break it into subfamilies [4,25,26,27]. Currently, this family is subdivided into: Sminthurinae Lubbock, 1862, with long antennae (mostly longer than the body), frequently lacking neosminthuroid chaetae on the parafurcal area and without cavities on the ungues, tibiotarsi with 8–9 chaetae on the distal (apical) whorl and 9–15 ventral (anterior) dental chaetae; Sphyrothecinae Betsch, 1980, with short antennae (smaller than the body), with a pair of neosminthuroid chaetae on the parafurcal area, tibiotarsi with 7 or more chaetae on the distal whorl, and dens with 6 or less ventral chaetae; and Songhaicinae Sánchez-García and Engel, 2016, with antennal size and ventral dental chaetotaxy variable, parafurcal area with or without neosminthuroid chaetae, tibiotarsi with six or fewer chaetae on the distal whorl and, more importantly, ungues with cavity and tunica, or only with a peculiar filament-like tunica [4,26,27,28]. The variety of morphologies gathered within Songhaicinae, with overlapping features also observed in the other two subfamilies, raises doubts about its validity, similarly to some intersecting morphology shared by some genera of Sminthuridae [4,27,29].

Here, we describe *Troglobentosminthurus* gen. nov., one of the most troglomorphic genus of Symphypleona regarding appendages elongation, from the Água Clara cave system, Caatinga domain, Bahia state, Brazil. We also compare it with other similar genera and provide notes on its morphology and the boundaries of Sminthuridae and Bourletiellidae Börner, 1913. Finally, we provide information on the species’ habitat, biology and threats.

## 2. Materials and Methods

Specimens were collected manually with entomological brushes and ethanol in the dark zones of Água Clara cave system (more details in topic 3.3, habitat and threats), preserved in 70% ethanol at 6 °C, cleared in Nesbitt’s solution, washed in Arlé’s liquid and mounted on glass slides in Hoyer medium, mixing [30,31] procedures. Ethanol-fixed specimens were photographed in a Nikon SMZ1500 stereomicroscope with a DS-Ri1 camera, under NIS-Elements AR v.4.51 software. Morphological studies and first drawings were made under Leica DM750 phase-contrast microscope, with an attached drawing tube. Final drawings were vectorized in Adobe Illustrator 2020 software. The type series was deposited at the Collection of Subterranean Invertebrates of Lavras (ISLA), linked to the Center for Studies in Subterranean Biology from the Federal University of Lavras (biologiasubterranea.com.br (accessed on 10 June 2020)).

The terminology used in the text and figures follows [32] to labial palp papillae and guard-chaetae, [33] to the labral chaetotaxy, [34] to the head and anterior large abdomen chaetotaxy, Vargovitsh system [35,36,37] with adaptations for the posterior large abdomen chaetotaxy, [38] for the small abdomen chaetotaxy, and [39] to part of the tibiotarsal chaetotaxy. Regarding the dens we considered the dorsal, dorso-internal and dorso-lateral rows summed as the dorsal chaetotaxy. Drawings and observations were made based on the entire type series.

The abbreviations used in the description and figures are: Abd.—abdominal segment(s); Ant.—antennal segment(s); Th.—thoracic segment(s). In the drawings, structures present or absent are marked with white arrows, and unpaired chaetae on the head and trunk are marked with a “*”. Ant. IV subsegments are counted from the base to apex. Head, trunk (thorax + abdomen) and furca description of the chaetotaxy is given by half body. Chaetae and labial papillae labels are marked in bold in the text.

## 3. Results

### 3.1. Taxonomic Summary and Subfamily Diagnosis

Order Symphypleona Börner, 1901 [40] *sensu* Bretfeld, 1986 [41]
Suborder Appendiciphora Bretfeld, 1986 [41]
Superfamily Sminthuroidea Bretfeld, 1994 [42]
Family Sminthuridae Lubbock, 1862 [43]
Subfamily Sminthurinae Lubbock, 1862 [43] *sensu* Bretfeld, 1999 [26]

*Diagnosis of the subfamily*. Antennae long, subequal or longer than the body, Ant. IV with 9–46 subsegments, Ant. II with or without 1 ventrolateral sensillum. Neosminthuroid chaetae mostly absent in the parafurcal area, with the exception of *Keratosminthurus*. Tibiotarsi distal whorl with 8–9 chaetae, pretarsi mostly with 2 chaetae, rarely with only the anterior chaeta, ungues without cavity or filament-like tunica. Dens with 9–15 ventral chaetae, mucro apically symmetrical, rarely asymmetrical (updated from [4,25,26,27]).

*Remarks*. Here, we complement Zeppelini et al. [4] diagnosis of the Sminthurinae due to some findings concerning the new genus and species. Bretfeld [26] pointed out that the Sminthuridae always have one pair of pretarsal chaetae, contrarily to the Bourletiellidae, which only present a single chaeta—the anterior one. However, at least *Varelasminthurus potiguarus* Silva, Palacios-Vargas and Bellini, 2015 [5], *Disparrhopalites naasaveqw* Bernard and Wynne 2017 [27] (both Songhaicinae), and now *Troglobentosminthurus luridus* gen. nov. sp. nov. (Sminthurinae) may have only the anterior pretarsal chaeta. We also observed the new species presents one ventro-lateral sensillum at the apex of the Ant. II, similar to a bothriotrichum, a feature described as a synapomorphy of the Bourletiellidae by Bretfeld [26]. However, at least *Stenognathriopes* (*Tenentiella*) *janssensi* Zeppelini and Silva 2012 [44] (Bourletiellinae Börner, 1913) does not have such morphology. It is worth noting that, although the pretarsal and antennal morphology of *Troglobentosminthurus luridus* gen. nov. sp. nov. resembles the Bourletiellidae, the new species belongs to Sminthurinae due to the absence of the Abd. V bothriotrichum **E** and of spatulate tenent-hairs on the tibiotarsi, and large abdomen bothriotricha **A**–**C** misaligned. Due to these observations, we included the absence of one pretarsal chaeta and the presence of an apical bothriotrichum-like sensillum on Ant. II as possible features also observed within the Sminthuridae (Sminthurinae), and not exclusive of the Bourletiellidae. Table 1 and the key below summarizes the main differences between the Sminthuridae subfamilies.

Key to the subfamilies of Sminthuridae Lubbock, 1862 [43]
1.Ungues with cavity and/or a filament-like tunica, tibiotarsal distal whorl with six or fewer chaetae…………………………………………………………………………………………………………………………………………………………………………………Songhaicinae Sánchez-García and Engel, 2016 [28] *sensu* Bernard and Wynne, 2017 [27]-.Ungues without cavity or a filament-like tunica, tibiotarsal distal whorl with 7–9 chaetae……………………………………22.Antennae shorter than the body, males’ Abd. VI with a pair of spines similar to the subanal appendages of the females, dens ventrally with 0–6 chaetae ………………………………………………………………………………………………………………………………………………………………………………………Sphyrothecinae Betsch, 1980 [25] *sensu* Bretfeld, 1999 [26]-.Antennae subequal or longer than the body, males’ Abd. VI without subanal appendages-like spines, dens ventrally with 9–15 chaetae………………………………………………………………………………………………………………………………………………………………………………………………………………Sminthurinae Lubbock, 1862 [43] *sensu* Bretfeld, 1999 [26]

### 3.2. Troglobentosminthurus gen. nov. Bellini, Medeiros and Souza

*Diagnosis of the genus.* Appendages remarkably elongated. Chaetae smooth and long, especially on antennae, tibiotarsi and posterior large abdomen, spine-like chaetae absent on head frontal area and dorsal large abdomen. Most chaetae alveoli slightly elongated, somewhat neosminthuroid-like, on the body and appendages. Ant. IV *Temeritas*-like, longer than the body combined with Ant. I–III, with 44 subsegments, Ant. II apically with one ventro-lateral bothriotrichum-like sensillum. Antennae and head chaetotaxy not sexually dimorphic. Mouthparts normal, not elongated, labial palp guard-chaeta **a1** laterally displaced and curved. Large abdomen without dorsal vesicles, small abdomen formed by Abd. V–VI. Bothriotricha **A**–**D** present and elongated, especially **D,** longer than the dens. Parafurcal area without sexually dimorphic or neosminthuroid chaetae. Trochanter III with a large spine in a rounded alveolus, tibiotarsi I–III distal whorls with 8/9/9 chaetae, respectively, pretarsi I–III with only a small anterior chaeta, ungues without cavity and tunica, but with underdeveloped pseudonychia, unguiculi without the apical filament. Ventral tube sacs warty, tenaculum with 1 + 1 distal chaetae. Dens ventral formula from the apex to the basis as 3, 2, 2, 1 … 1, mucro without chaeta, with the inner lamella serrated and the outer smooth, mucronal apex symmetric.

*Etymology.* The new genus honors our dear friend, the emergent skillful biospeleologist Dr. Diego de Medeiros Bento, nicknamed “Troglobento”. Diego works at the National Center for Cave Research and Conservation (ICMBio/CECAV), tracking, mapping and studying caves and cave fauna in Brazil, mainly in the semiarid region of Rio Grande do Norte state, Brazil. His efforts, as well as those of many other professionals working for CECAV and other entities, helped to reveal and preserve several Brazilian caves. Contrary and irresponsibly, the Brazilian president signed in 2022 the federal decree 10,935, which enables the destruction of even the most relevant caves in Brazil for commercial purposes, putting at risk such efforts. Thus, the new genus honors not only Dr. Diego Bento but also many other Brazilian professionals committed to the conservation of underground environments.

*Remarks.* The overall morphology of the new genus puts it within the *Temeritas*-group *sensu* Medeiros et al. [29], which gathers *Temeritas* Richards, 1963 in [51], *Richardsitas* Betsch, 1975 [52], *Galeriella* Ćurčić and Lučić, 2007 in [53], and *Keratosminthurus*. This group of genera and *Troglobentosminthurus* gen. nov. share: antennae mostly longer than the body, Ant. IV with 18–46 subsegments, post-antennal chaeta absent and metatrochanteral spine present. The Ant. IV with 44 subsegments, presence of eyes, head without sexually dimorphic features, large abdomen lacking spines but with slender chaetae, parafurcal area without neosminthuroid chaetae and tibiotarsi without capitate tenent-hairs put the new genus closer to *Temeritas stricto sensu* Medeiros and Bellini [54]. However, *Temeritas* differs from *Troglobentosminthurus* gen. nov. especially in the frontal area of the head with spines (vs. absent), unguiculi with the apical filament (vs. without), dens ventrally with 13 chaetae (vs. 9), specimens pigmented, mostly with complex color patterns (vs. lacking pigments), eyes 8 + 8 and normally developed (vs. 5 + 5 reduced eyes) [54]. The highly troglomorphic profile of *Troglobentosminthurus* gen. nov. strongly resembles that of *Galeriella*, a monotypic cave genus only recorded from Bosnia and Herzegovina [53,55], but *Galeriella* differs from *Troglobentosminthurus* gen. nov. in Ant. IV with 32 subsegments (vs. 44), eyes absent (vs. 5 + 5), ungues without teeth and pseudonychia (vs. present), tenaculum without chaetae (vs. 1 + 1) and mucro with both lamellae serrated (vs. only one). The distribution supports these genera emerged independently as well, since *Galeriella* was only found in the Balkan Peninsula, Palaearctic Region, and the new genus in the southern region of Caatinga Domain, Neotropical Region. *Galeriella* also presents a remarkably underdeveloped chaetotaxy, different from most Sminthurinae, including the *Temeritas*-group, which suggests that the two type specimens, listed as males, may be juveniles, or at least that some chaetae at the proximal antennal articles, head, tibiotarsi and ventral dens may have been overlooked in the original description [53]. Such doubts prevent us from better comparing the genus with *Troglobentosminthurus* gen. nov. Further comparisons between the *Temeritas*-group genera are presented in Table 2 and the identification key below.

Although the antennal elongation of *Troglobentosminthurus* gen. nov. is supposedly linked to the cave life, it may also be the result, at least partially, of a shared ancestral with *Temeritas*, which also have long and subsegmented Ant. IV, but live in epigeic ecosystems [54]. Morphology, as previously discussed, and distribution support this hypothesis. There are currently 12 species of Neotropical *Temeritas*, with seven of them registered from Brazil, of which three were also found in the Caatinga domain [2,54]. Nevertheless, only a phylogenetic study could test if *Troglobentosminthurus* gen. nov. and *Temeritas* are closely related. 

The elongation of the tibiotarsi in the new genus splits the chaetae on each whorl following Nayrolles [39], suggesting at first that the distal whorl is similar to the Songhaicinae with only five or six chaetae. However, a more detailed study of the homology of the tibiotarsal chaetotaxy shows they fit the diagnosis of Sminthurinae, with 8/9/9 chaetae on tibiotarsi I–III, respectively, and three chaetae occur on the upper region of the distal tibiotarsi (see more details in the description of the new species). Other peculiar details on the morphology of the new genus are: the labial palp papillae similar to that of *Sminthurus* Latreille, 1802 [56] and *Disparrhopalites naasaveqw*, with the **a1** guard-chaeta large, apically curved and laterally displaced, almost or clearly external to the papilla **C** [27,32], a trait not reported to any *Temeritas*, *Richardsitas* or *Keratosminthurus* species (unknown to *Galeriella*) [4,29,54]; the absence of the posterior pretarsal chaeta, only recorded before within Sminthuridae to the Songhaicinae genera *Varelasminthurus* and *Disparrhopalites* Stach, 1956 [5,27,57]; and the Ant. II apically with one ventro-lateral bothriotrichum-like sensillum, a feature previously only observed in the Bourletiellidae [26].

It is important to state we did not consider any chaetae on the parafurcal area as neosminthuroid because they all have similar slightly elongated alveoli, in accordance with most chaetae seen on the appendages and body. Further details on the chaetae which present different alveoli are detailed in the description of the new species. 

Key to the genera of *Temeritas*-group *sensu* Medeiros et al. [29].
1.Eyes and ungual inner teeth absent……………………………………………………………………………………………………………………………………………………………………………………………………………………………………………………*Galeriella* Ćurčić and Lučić, 2007 in [53]-.Eyes and ungual inner teeth present……………………………………………………………………………………………………22Clypeus and Ant. III with sexually dimorphic chaetae on males, parafurcal area with a pair of neosminthuroid chaetae……………………………………………………………………………………………………………*Keratosminthurus* Zeppelini, 2020 in [4]-.Clypeus and Ant. III without sexually dimorphic chaetae, parafurcal area lacking neosminthuroid chaetae…………………33.Specimens pale, eyes 5 + 5, head frontal area without spines, pretarsi posterior chaeta absent, dens ventrally with nine chaeta……………………………………………………………………………………………………………………………………………………………………………………………………………………………………………………………*Troglobentosminthurus* gen. nov.-.Specimens pigmented, eyes 8 + 8, head frontal area with spines, pretarsi posterior chaeta present, dens ventrally with 13 chaetae…………………………………………………………………………………………………………………………………………………………………………………………………………………………………………………………………………………………………44.Males with dimorphic short candle-shaped spines on the dorsal abdomen, capitate tenent-hairs present on tibiotarsi II–III………………………………………………………………………………………………………………………………………………………………………………………………………………………………………………………*Richardsitas* Betsch, 1975 [52]-.Specimens without sexually dimorphic spines on the dorsal abdomen, tenent-hairs acuminate……………………………………………………………………………………………………………………………………………………*Temeritas* Richards, 1963 in [51]

### 3.3. Troglobentosminthurus luridus gen. nov. sp. nov. Souza, Medeiros and Bellini (Figures 1–10 and Table 2)

**Figure 1:**
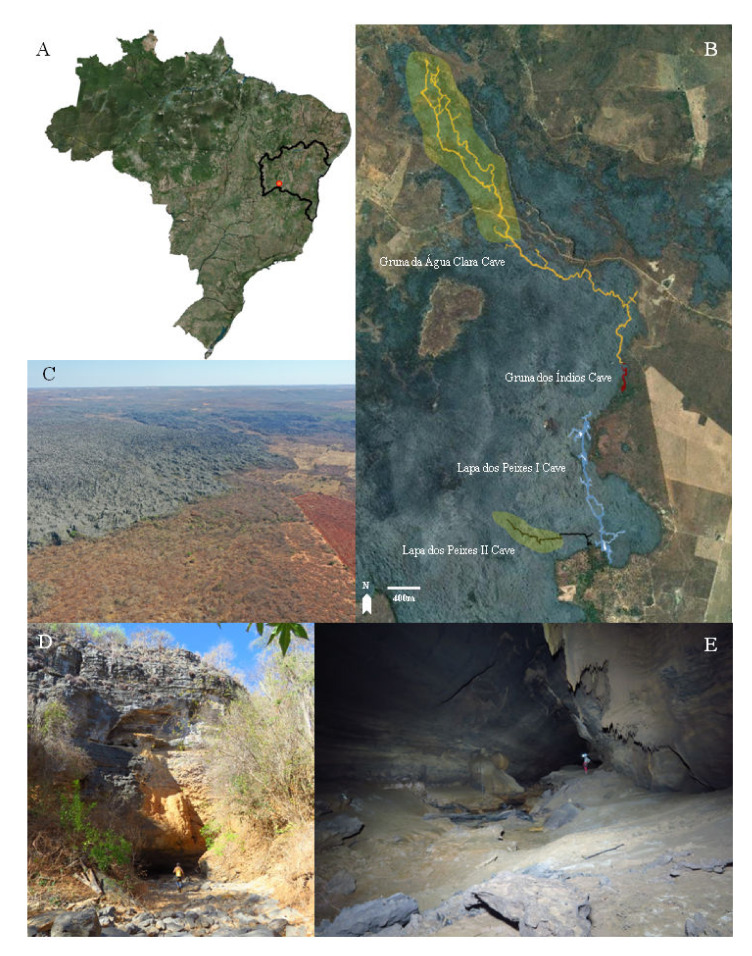
Type locality of *Troglobentosminthurus luridus* gen. nov. sp. nov.: (**A**) Água Clara cave system (ACCS) location, in the municipality of Carinhanha, Bahia state, Brazil; (**B**) spatial distribution of the caves within ACCS, the areas in translucent yellow correspond to the regions where the specimens of the new species were found; (**C**) aerial view from the surrounding region of the ACCS; (**D**) one of the entrances of the Gruna da Água Clara cave, which corresponds to a sink; (**E**) inner conduit of the Gruna da Água Clara cave.

*Type material*. Holotype: female on slide, Serra do Ramalho, Água Clara cave system, Carinhanha municipality, Bahia state, Brazil (13°47′09.383″ S, 42°06′07.667″ W, Figure 1), 11/X/2017, R.L. Ferreira col (ISLA 79664). Paratypes: three males and one female, same data of the holotype, except for one male, collected on 16/X/2017 (ISLA 79664 and 79662).

*Diagnosis of the species.* Specimens pale, without pigments, except for orange eyepatches. Dorso-frontal area with a cuticle crest on each side of the head posterior to the eyes, similar to a suture. Head chaetotaxy with only two medial unpaired chaetae, one frontal and one clypeal. Eyes 5 + 5, reduced in size. Trochanter III with five regular chaetae; tibiotarsi I–III with five oval organs each. Ungues with 3–4 inner teeth, unguiculi truncate with the inner lamella proximally serrated. Large abdomen of males with a dimorphic row of four chaetae upper to the parafurcal area (completely absent in females). Females’ subanal appendage smooth and apically bent. Dens with 27 dorsal chaetae; mucro sexually dimorphic, with large crenulations on males in the serrated edge. 

**Figure 2:**
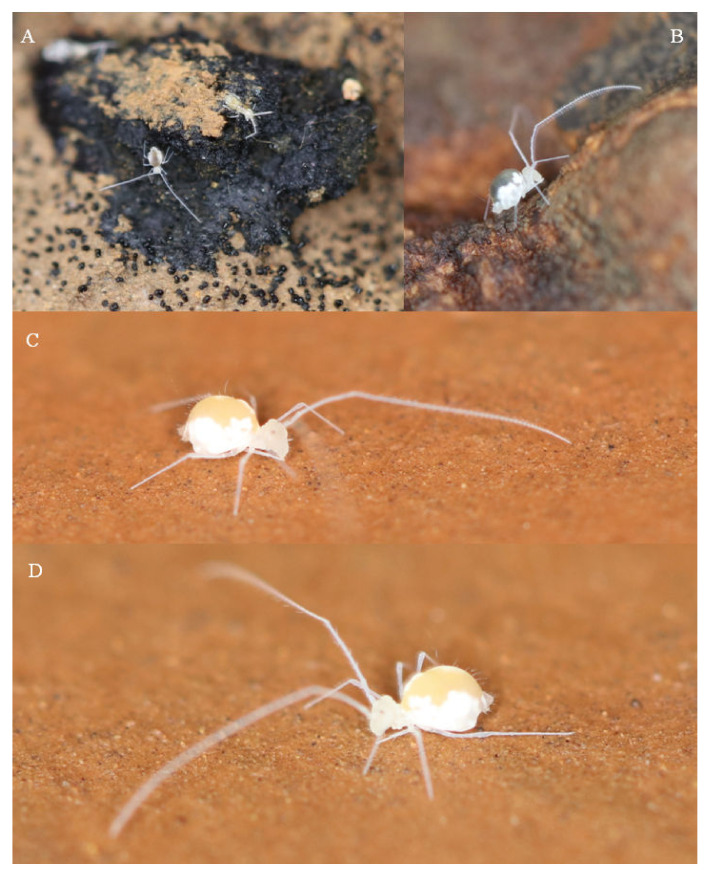
Living specimens of *Troglobentosminthurus luridus* gen. nov. sp. nov.: (**A**) one specimen apparently feeding on a bat guano pile in the Lapa dos Peixes II cave (another springtail specimen in the same pellet); (**B**) specimen walking around a dry bat guano pile in the Lapa dos Peixes II cave; (**C**,**D**) specimens freely walking on the cave muddy sediment in the deeper portion of the Gruna da Água Clara cave.

**Figure 3:**
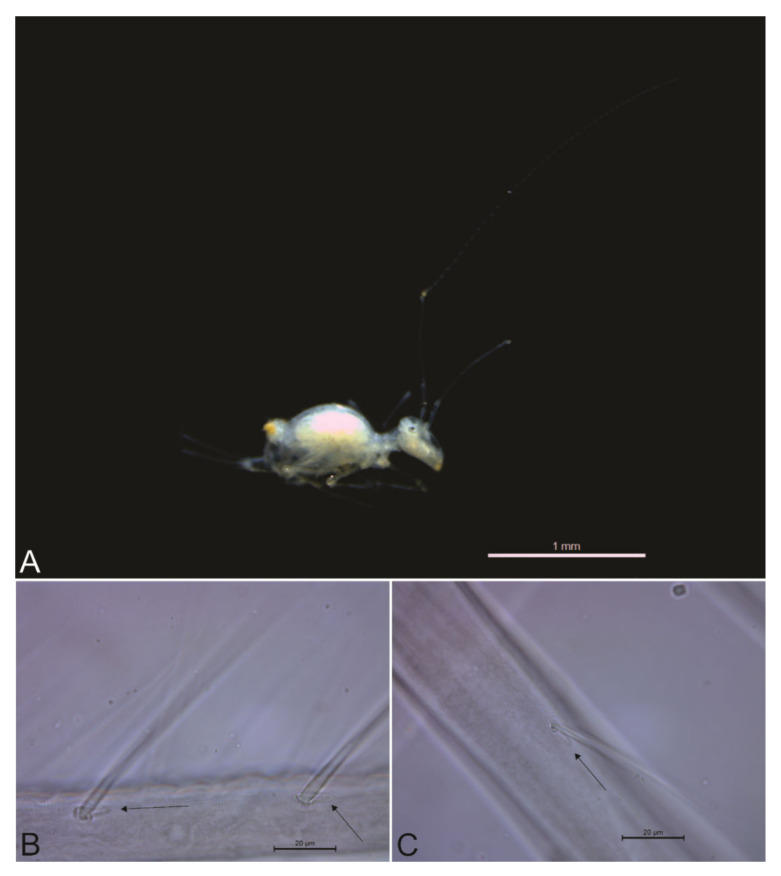
*Troglobentosminthurus luridus* gen. nov. sp. nov. morphology: (**A**) habitus of a fixed specimen in ethanol; (**B**) two chaetae elongate alveoli on the dorsal dens (marked with black arrows); (**C**) one chaeta elongate alveolus on the tibiotarsus II (marked with a black arrow).

*Description.* Body (head + trunk) length of the type series ranging between 1.15 and 1.68 mm, holotype with 1.68 mm, males average size 1.21 mm, females average size 1.63 mm, entire type series average size 1.38 mm. Body color of live specimens pale white, with a translucent yellowish dorsal large abdomen, pigments absent with the exception of small orange eyepatches (Figure 2). Fixed specimens also pale, with dark eyepatches (Figure 3A). Antennae, head, dorsal thorax and abdomen, legs and dorsal furca with long slender acuminate smooth chaetae inserted in slightly elongated alveoli (Figure 3B,C). Spines or spine-like chaetae absent, even on the head frontal area and dorsal large abdomen. 

**Figure 4:**
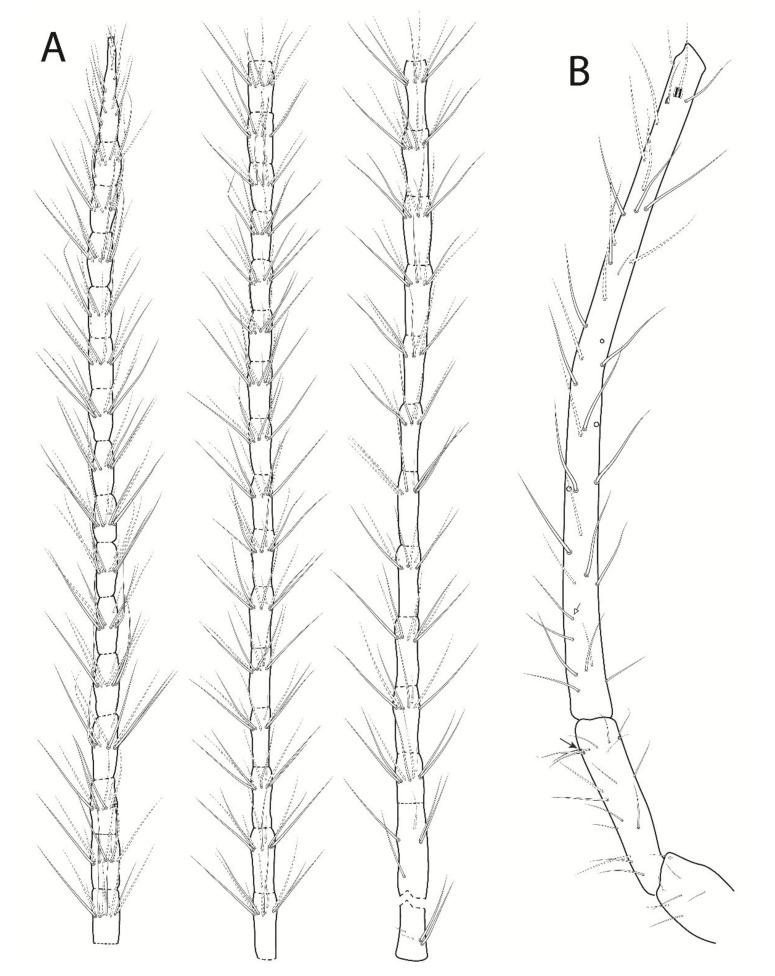
*Troglobentosminthurus luridus* gen. nov. sp. nov. left antenna: (**A**) Ant. IV subsegments, from left to right the more apical to the more proximal ones (**left**, 29–44 subsegments; **middle**, 13–28; **right** 1–12); (**B**) Ant. I–III, white arrow marks a chaeta present or absent, black arrow marks the ventro-lateral bothriotrichum-like sensillum.

**Figure 5:**
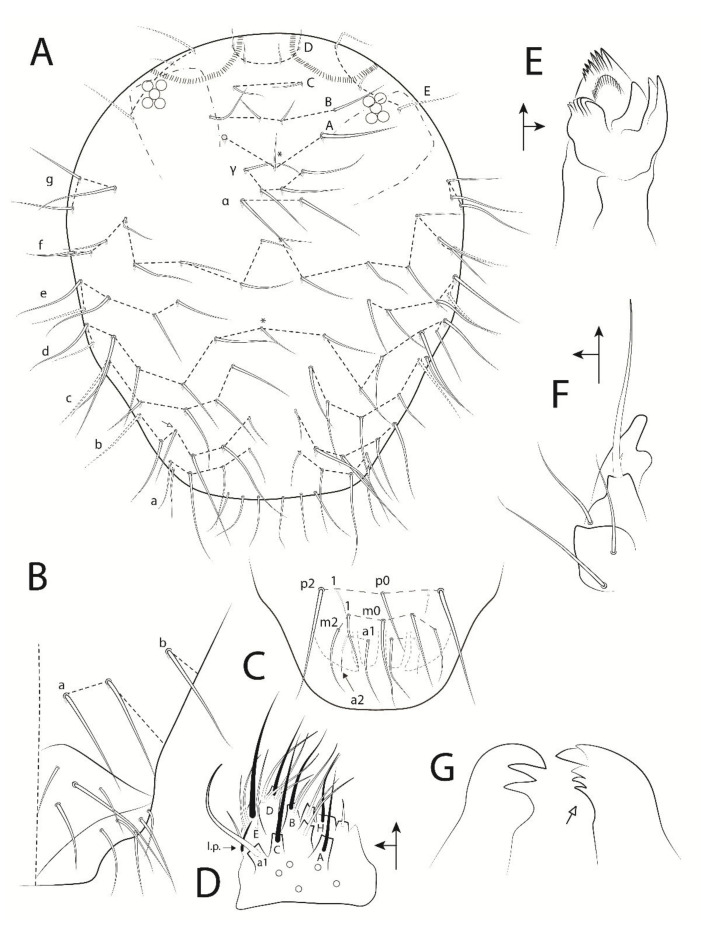
*Troglobentosminthurus luridus* gen. nov. sp. nov. head: (**A**) anterior head chaetotaxy, Ant. I marked in dotted lines, white arrow marks a chaeta present or absent, asterisks mark unpaired central chaetae; (**B**) right side of the posterior (ventral) head chaetotaxy, including post-labial, labial basomedial and basolateral chaetae; (**C**) labral chaetotaxy; (**D**) left labial palp; (**E**) right maxilla capitulum; (**F**) left maxillary outer lobe and sublobal plate; (**G**) incisive (apical) teeth of the mandibles, white arrow marks a tooth in the right mandible which can be absent.

Head (Figure 4 and Figure 5). Antennae more than twice the length of the body, with 3.65 mm in the holotype. Holotype antennal segments ratio I:II:III:IV as 1:2.6:10:37. Ant. IV longer than the Ant. I–III + body, with 44 subsegments (n = 1, only holotype with a complete antenna); subsegment 1 with at least nine chaetae (subsegment broken), two proximal reduced, subsegment 2 with eight chaetae, 3 with seven chaetae, 4 with eight chaetae, 5–7 with eight chaetae each, 8 with nine chaetae, 10–11 with 10 chaetae each, 12 with nine chaetae, 13–15 with 10 chaetae each, 16–17 with nine chaetae each, 18–43 with 10 chaetae each, and subsegment 44 with 20 chaetae (Figure 4A). Ant. III with 38–39 chaetae, apical organ sensory rods in two independent shallow invaginations, surrounding subapical microsensillum with rounded apex (Figure 4B). Ant. II with 18 chaetae, one of them as a ventro-lateral bothriotrichum-like sensillum (Figure 4B). Ant. I with seven chaetae (Figure 4B). Head length (eyes to mouth) of the holotype 0.55 mm. Eyes 5 + 5, reduced in size, apparently with an extra vestigial lens on each eyepatch. Clypeal area **a**–**g** series with 7/6–7/6/6(+1)/5/8/3 chaetae, respectively (Figure 5A,B). Interantennal area with only **α** and **γ** series with one and two chaetae, respectively, near to **g** series central field; frontal area **A**–**E** series with 1(+1)/2/1/2/3 chaetae, respectively; dorso-frontal area with a cuticle crest on each side of the head posterior to the eyes, similar to a suture; interocular chaetae absent (Figure 5A). Ventral head with two **a** and one **b** chaetae on the post-labial region, labial basomedian field with four chaetae, one of them large, and basolateral field with five chaetae (Figure 5B). Six prelabral chaetae present (Figure 5A), labral **a**, **m**, **p** series with 2, 2(+1), 2(+1) chaetae respectively, **p1** smaller and thinner than the others, **m1** smaller than **m0**, **a2** reduced, and **p2** larger than the others (Figure 5C). Labial palp with five proximal chaetae, papillae formula of guard-chaetae for each papilla as **H**(2), **A**(0), **B**(5), **C**(0), **D**(4), **E**(4) + lateral process (**l**.**p**.) not reaching the papilla **E** base, papilla **H** guard-chaetae heterogeneous, papilla **B** guard-chaeta **a1** laterally displaced to **C**, large and curved anteriorly (Figure 5D). Maxilla with three lateral teeth and three central large lamellae (Figure 5E). Maxillary outer lobe developed, apical chaeta smooth with a proximo-internal barb (or toothlet), basal chaeta smaller than the apical one, oral fold with two chaetae, sublobal plate internally folded, without chaeta-like appendages (Figure 5F). Mandibles asymmetrical with 3 + 4–5 incisive apical teeth (Figure 5G).

**Figure 6:**
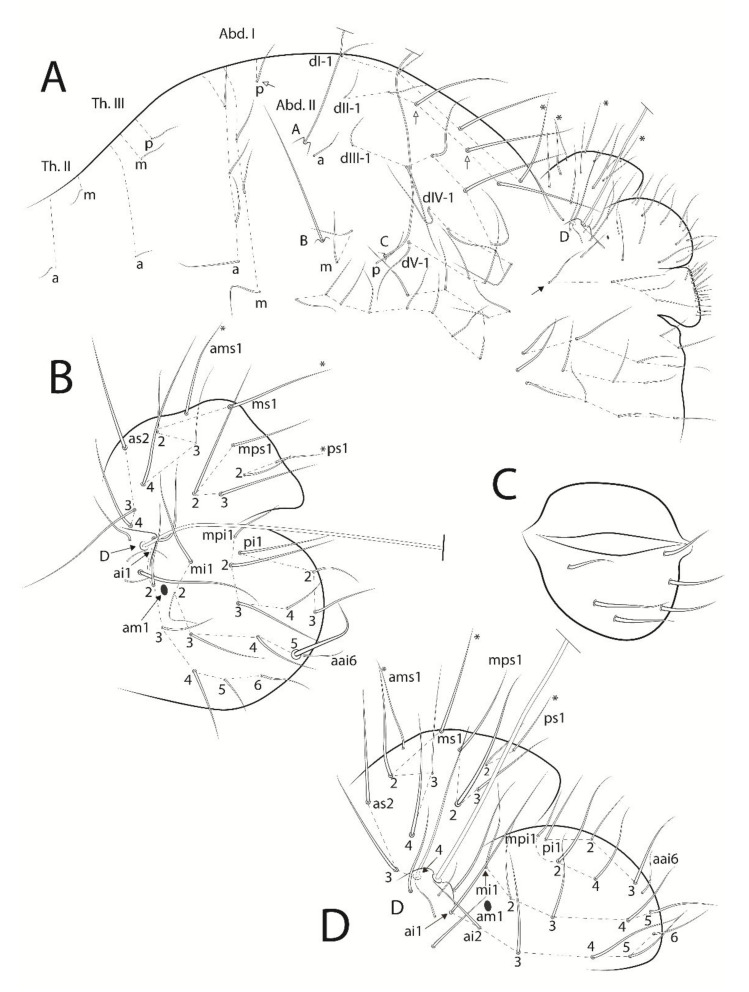
*Troglobentosminthurus luridus* gen. nov. sp. nov. trunk: (**A**) large and small abdomens of the male, white arrows mark chaetae present or absent, asterisks mark unpaired central chaetae, black arrow marks a row of chaetae only seen in males; (**B**) female’s small abdomen, asterisks mark unpaired central chaetae; (**C**) female’s genital plate; (**D**) male’s small abdomen in detail, asterisks mark unpaired central chaetae.

Trunk (Figure 6). Trunk length of the holotype 1.13 mm. Large abdomen: thorax continuous with abdomen without any segmentation, constrictions or vesicles in both sexes. Sexual dimorphism on the large abdomen restricted to a row with four chaetae present only on males above the parafurcal area (Figure 6A, black arrow). Chaetotaxy as: Th. II with one **a** and one **m** chaetae; Th. III with one **a**, one **m** and one **p** chaetae; Abd. I with four **a**, three **m** and 0–1 **p** chaetae; bothriotricha **A**, **B** and **C** present and elongated on Abd. II and misaligned; bothriotrichia **A** with one (**a**), **B** with two (**m**) and **C** with one (**p**) accessory chaetae, respectively; Abd. III–IV with five main series of chaetae above the bothriotrichum **C**: **dI-1** with 3(+1), **dII-1** with 3–5, **dIII-1** with five, **dIV-1** and **dV-1** with three chaetae each, plus two ventro-lateral rows of chaetae under the bothriotrichum **C,** with four and eight chaetae, respectively. Parafurcal area with four rows of chaetae with two, four, four, four (total of 14) chaetae on males and females, all chaetae with the same type of elongate alveoli, neosminthuroid chaetae absent (Figure 6A). Small abdomen: in both sexes including Abd. V and bothriotrichum **D**, the latter longer than the dens, **E** absent (Figure 6A,B,D). Female’s dorsal anal valve with **as2–4**, **ams1**, **ms1**–**4**, **mps1–3** and **ps1–2** chaetae, **ams1**, **ms1** and **ps1** unpaired; each ventral anal valve with **aai6**, **ai1–6**, **mi1–5**, **mpi1–4** and **pi1–3** chaetae, **am1** as an oval organ and **mi5** as the subanal appendage, slender, smooth and curved toward the anus opening (Figure 6B). Genital plate of the female with 3 + 3 chaetae on the ventral field (Figure 6C). Male’s dorsal anal valve with **as2–3**, **ams1**, **ms1**–**4**, **mps1–3** and **ps1–2** chaetae, **ams1**, **ms1** and **ps1** unpaired; each ventral anal valve with **aai6**, **ai1–6**, **mi1–5**, **mpi1–2, mpi4** (**mpi3** absent) and **pi1–3** chaetae, **am1** as an oval organ (Figure 6D). Genital plate of the male with about 13 + 1 + 13 chaetae (Figure 6A).

**Figure 7:**
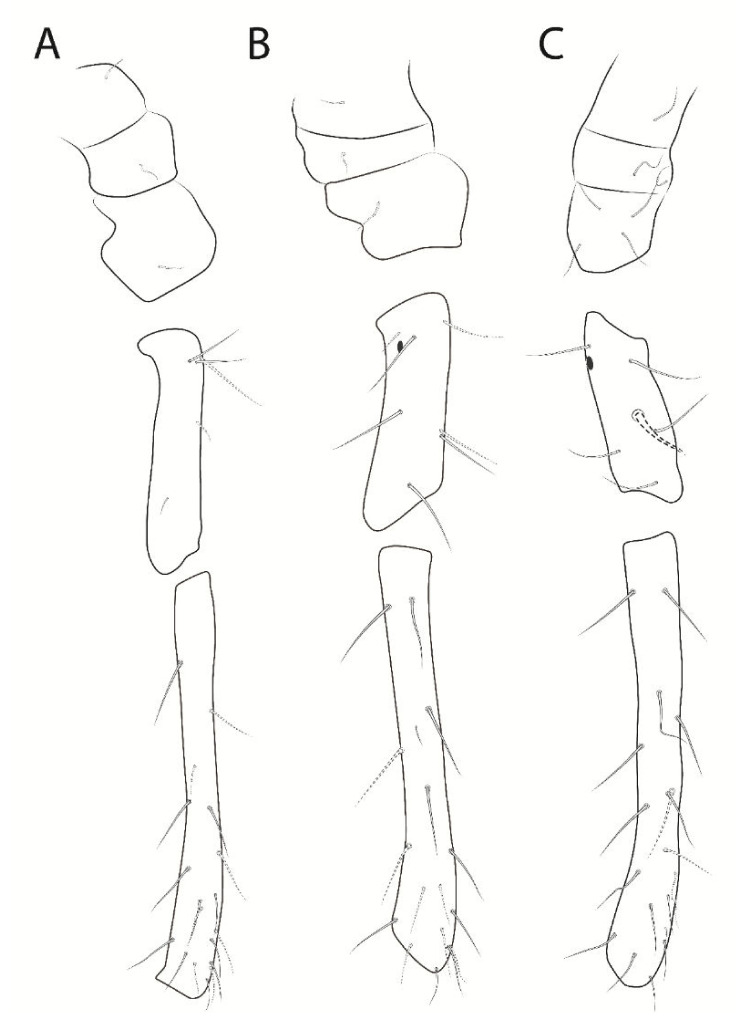
*Troglobentosminthurus luridus* gen. nov. sp. nov. legs: (**A**) epicoxa to femur of leg I; (**B**) epicoxa to femur of leg II; (**C**) epicoxa to femur of leg III.

**Figure 8:**
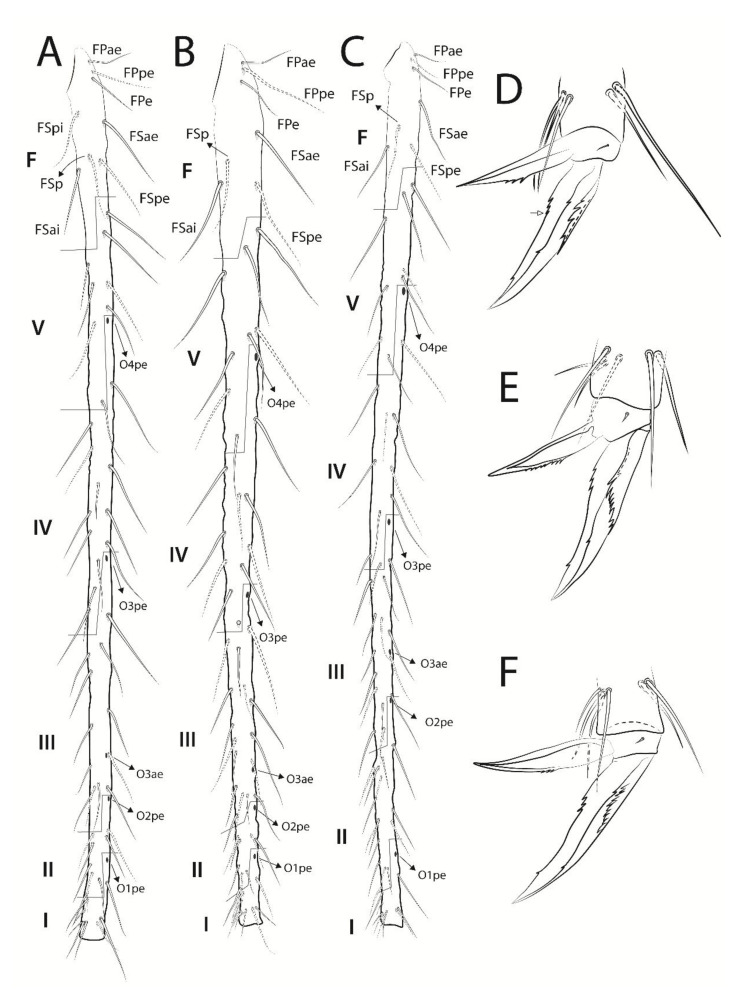
*Troglobentosminthurus luridus* gen. nov. sp. nov. legs: (**A**) tibiotarsus I; (**B**) tibiotarsus II; (**C**) tibiotarsus III; (**D**) empodial complex I, anterior view, white arrow marks a ungual tooth which can be absent; (**E**) empodial complex II, anterior view; (**F**) empodial complex III, anterior view.

Legs (Figure 7 and Figure 8). Leg I: epicoxa, subcoxa and coxa with one chaeta each; trochanter with five chaetae, two of them reduced; femur with 18 chaetae, one of them reduced; tibiotarsus with 56 chaetae and five oval organs (**O1pe**, **O2pe**, **O3ae**, **O3pe**, **O4pe**) on the dorsal side, proximal group **F** with eight chaetae (**FPae**, **FPpe**, **FPe**, **FSae**, **FSpe**, **FSpi**, **FSp** and **FSai**), distal whorl with seven chaetae plus one long acuminate tenent-hair, tibiotarsal groups of chaetae and whorls spaced due to the elongation of the segment (Figure 7A and Figure 8A); tibiotarsus I length in the holotype 0.76 mm. Leg II: epicoxa, subcoxa and coxa with one chaeta each; trochanter with seven chaetae, one of them reduced, plus one proximal oval organ; femur with 17 chaetae, one of them reduced; tibiotarsus with 57 chaetae and five oval organs (**O1pe**, **O2pe**, **O3ae**, **O3pe**, **O4pe**) on the dorsal side, proximal group **F** with seven chaetae (**FPae**, **FPpe**, **FPe**, **FSae**, **FSpe**, **FSp** and **FSai**), distal whorl with eight chaetae plus one long acuminate tenent-hair, tibiotarsal groups of chaetae and whorls spaced due to the elongation of the segment (Figure 7B and Figure 8B); tibiotarsus II length in the holotype 0.69 mm. Leg III: epicoxa with one, subcoxa with two and coxa with four chaetae, respectively; trochanter with five regular chaetae and a thick trochanteral spine within a rounded alveolus, plus one proximal oval organ; femur with 19 chaetae; tibiotarsus with 62 chaetae and five oval organs (**O1pe**, **O2pe**, **O3ae**, **O3pe**, **O4pe**) on the dorsal side, proximal group **F** with seven chaetae (**FPae**, **FPpe**, **FPe**, **FSae**, **FSpe**, **FSp** and **FSai**), distal whorl with eight chaetae plus one regular-sized acuminate tenent-hair, tibiotarsal groups of chaetae and whorls spaced due to the elongation of the segment (Figure 7C and Figure 8C); tibiotarsus III length in the holotype 0.85 mm. Foot complexes: pretarsi I–III with one discrete anterior chaeta each, posterior chaeta absent; ungues I–III with two pairs of lateral teeth, internal lamella with three proximal (2–3 on unguis I) and one apical smaller teeth, dorsal tooth absent, ungues without tunica but with partially developed pseudonychia; unguiculi I–III with proximally serrated antero-internal lamella, unguiculi I and III with about three serrations, II with about eight, postero-external lamella smooth, unguiculus filament absent in all legs (Figure 8D–F).

**Figure 9:**
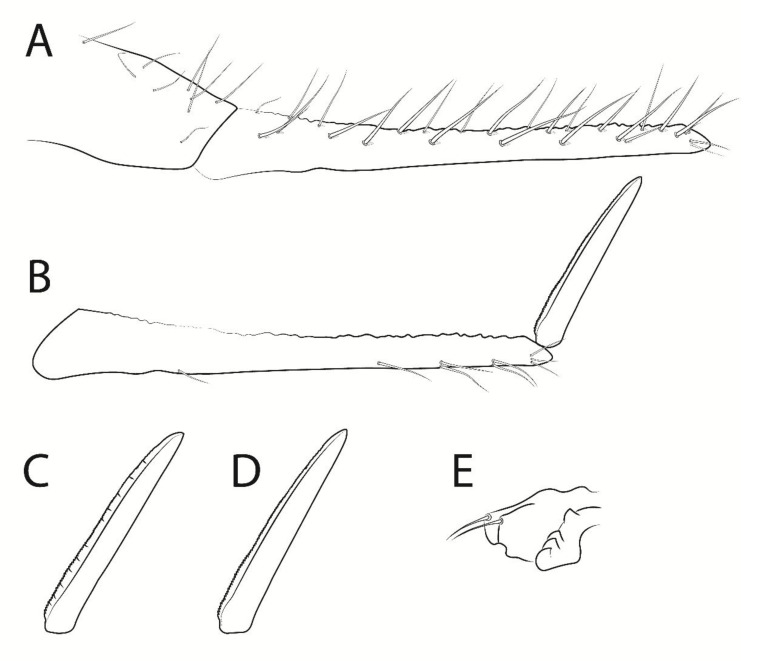
*Troglobentosminthurus luridus* gen. nov. sp. nov. furcula: (**A**) dorso-lateral view of the manubrium and dens; (**B**) ventro-lateral view of the left dens and mucro of a female; (**C**,**D**) sexual dimorphism regarding the mucro: (**C**) lateral view of the left mucro of a male; (**D**) lateral view of the left mucro of a female; (**E**) tenaculum.

Abdominal appendages (Figure 9). Ventral tube with 1 + 1 chaetae on the corpus and 1 + 1 on the lateral flaps, sacs long and warty. Manubrium with nine, and dens with 27 dorsal chaetae, respectively (Figure 9A), dens ventrally (anteriorly) with nine chaetae inside rounded alveoli, following the formula from the apex to the basis: 3, 2, 2, 1 … 1 (Figure 9B). Mucro with the inner lamella slightly serrated with about 70 small teeth, outer lamella smooth; mucro sexually dimorphic, with large crenulations on males in the serrated edge; mucronal chaeta absent (Figure 9B–D). Ratio mucro:dens:manubrium of the holotype as 1:4.6:1.8. Tenaculum with three distal teeth plus one proximal basal tubercle on each ramus, corpus with 1 + 1 apical chaetae (Figure 9E).

*Etymology*. The new species was named *luridus* (from Latin: lurid or shockingly macabre) due to its pale, ghostly habitus, and due to one of the type-series females has remnants of a devoured male on its guts, suggesting the species can be cannibalistic or necrophile.

*Habitat and threats*. Specimens of *Troglobentosminthurus luridus* gen. nov. sp. nov. were found in two caves from the Água Clara cave system (ACCS), located in the karst region of Serra do Ramalho, Carinhanha municipality, Bahia state, Brazil (Figure 1A). The ACCS is composed of four limestone caves (Gruna da Água Clara, Gruna dos Índios, Lapa dos Peixes I and Lapa dos Peixes II caves) which are trespassed by an intermittent stream, active during the austral summer (October until March) (Figure 1B). The whole system presents around 24 km, but there are still unsurveyed galleries. According to Köppen’s climate classification system, the local climate is “Aw”, with dry winter and an average annual rainfall of 640 mm [59]. The Serra do Ramalho region is inserted in the Caatinga domain (an exclusive Brazilian semiarid biome), with transitional areas to the Cerrado (Brazilian Savanna) [60].

Specimens of *Troglobentosminthurus luridus* gen. nov. sp. nov. were only found in deep and moistened areas of the dark zone of the system, which have only horizontal caves. It is interesting noting that the two caves located at the intermediate portion of the ACCS (Gruna dos Índios and Lapa dos Peixes caves) have the main conduit quite dry (even with the presence of some ponds) due to the wind, which trespasses both caves. Such caves present entrances on both sides of their main conduit, thus favoring the airflow. Hence, individuals of *Troglobentosminthurus luridus* gen. nov. sp. nov. were not observed in this intermediate portion of the ACCS, probably due to the unstable climatic conditions of the caves.

The individuals were found both associated with small bat guano pellets (especially in the Lapa dos Peixes II cave, Figure 2A,B) but also freely walking on the muddy sediment in the deep areas of the Gruna da Água Clara cave (Figure 2C,D). In the latter, specimens were often found in areas with no visible organic debris, so it is likely that they might be feeding on some microbial content associated with the cave sediments. It is interesting to highlight that the caves of ACCS are seasonally flooded, as strong water flows trespass the caves during the rainy period, coming from several entrances along the ACCS (Figure 1D). Thus, individuals of *Troglobentosminthurus luridus* gen. nov. sp. nov. somehow overcome such floodings, probably by climbing up the cave’s walls, which can be considerably high (Figure 1E), or entering rock cracks, but this subject certainly merits further research.

It is also noteworthy that in the deep regions where specimens are found, there are almost only troglobitic species [61], thus indicating that only highly specialized species can thrive under the restrictive conditions occurring in such areas (especially strong oligotrophy). Among the 30 cave-restricted species observed in the ACCS, only 8 (~26%) are formally described [61]. Thus, it is of paramount importance to describe the remaining troglomorphic species in order to preserve the ACCS, since non-described species are often ignored in conservation actions [62].

The Serra do Ramalho region has been the target of increasing deforestation in the last decades, mainly due to charcoal production and the establishment of monocultures (Figure 1C). Forest removal in the areas that directly influence the caves can alter the organic sediments supply to the caves and negatively impacts bat communities as well, thus affecting the food resources available for the hypogean fauna [61]. Furthermore, species associated with systems with low availability of guano and carcasses, such as the ACCS, may be more dependent on allochthonous food resources [61].

Considering the recent setbacks in the Brazilian laws regarding cave protection [63], it is urgent to describe cave-restricted species from an important hotspot of subterranean biodiversity as the Agua Clara cave system. Since the caves are under serious threat, it is more urgent than ever to formally describe such endemic species, as they might become the only effective protection for the habitats they rely on.

*Biology*. The overall morphology of *Troglobentosminthurus luridus* gen. nov. sp. nov. with extraordinarily long appendages and chaetae, loss of pigments and part of the eyes, as well as the reduction of the eye lenses, support the species is a troglobite, especially compared to other species of the *Temeritas*-group. As discussed before, the limited distribution of the species within the ACSS, only found in deeper areas, reinforces this hypothesis.

**Figure 10:**
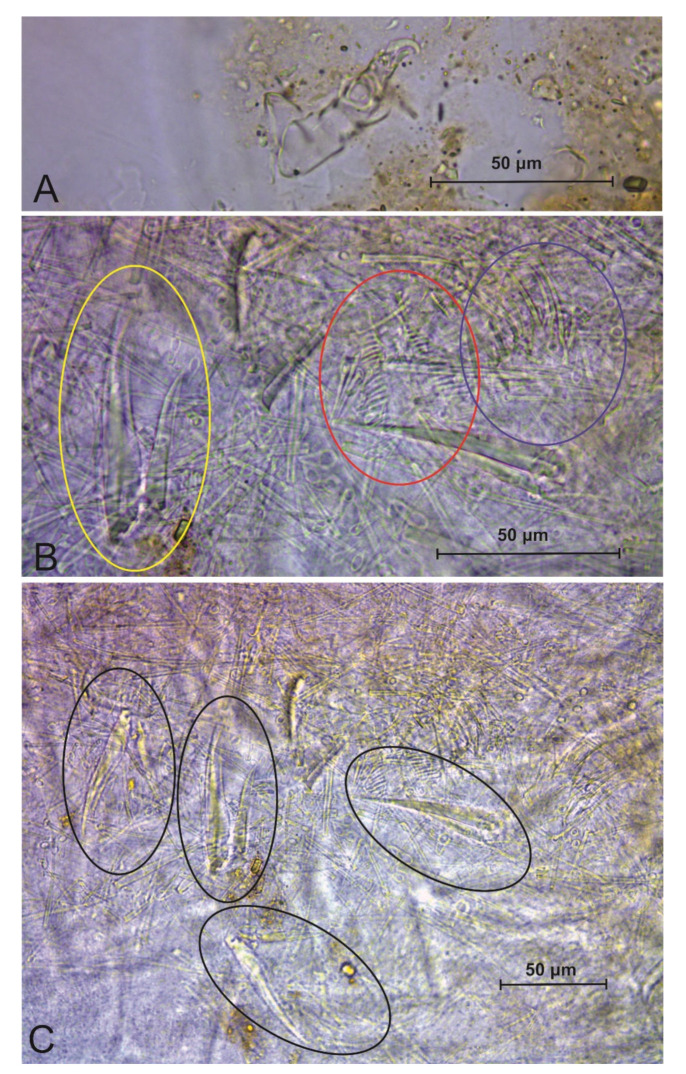
*Troglobentosminthurus luridus* gen. nov. sp. nov., gut contents: (**A**) a male’s contents, with a mite leg at the center and dark matter mixed with soil particles at the right side; (**B**) holotype (female) contents, yellow circle marks an empodial complex, red marks the testicles, and blue marks one labial palp; (**C**) holotype (female) contents, black circles mark four devoured empodial complexes.

The type specimens have soil particles in the guts mixed with some dark matter, suggesting the specimens may feed on bat guano, cave sediments with microbes or particles of organic matter (Figure 10A). However, the gut contents of two specimens were more unusual. One of the studied males has two mite legs in its contents (Figure 10A), while the holotype, a female, has the remains of one male of the same species in its digestive system (Figure 10B,C). In the case of the analyzed male paratype, we could not track any other clear part of the mite other than the legs, but in the case of the female, we could confirm the morphology of the six empodial complexes of the digested specimen as the ones described to *Troglobentosminthurus luridus* gen. nov. sp. nov. Although this devoured specimen was fragmented, we could also identify its testicles, both labial palps and many body chaetae on the remains found inside the holotype, supporting it is a male of her own species (Figure 10B,C). In this scenario *Troglobentosminthurus luridus* gen. nov. sp. nov. specimens may be occasional predators/cannibals, or even necrophiles and feed on dead arthropods. Such uncommon feeding habitats for springtails must be better studied to confirm which hypothesis fits best the biology of the new species. However, under extreme oligotrophic conditions, such as those observed in the areas where the specimens are found inside the ACCS, all raised behaviors (predation/cannibalism/necrophagy) are quite plausible.

*Remarks.* Since the genus is monotypic, the species and genus diagnoses should be considered complementary. They also may have conflicting characters, as specific traits marked as diagnostic of the genus and vice-versa, which should be better understood with the description of further species of *Troglobentosminthurus* gen. nov. in the future, if any. For taxonomical comparisons, see the remarks of the new genus and Table 2.

## 4. Discussion

The description of *Troglobentosminthurus* gen. nov., as well as *Keratosminthurus* and the Songhaicinae, test the boundaries of the internal systematics of the Sminthuroidea. Until now, no representative molecular analysis aiming to resolve the internal relationships of the Symphypleona was published. Recent studies using different markers and phylogenetic methods only gathered a few taxa of the order, unable to clearly investigate the validity and affinities of its internal groups, as in [64,65,66,67,68,69]. In this scenario, arguably, the most reliable phylogenetic data available are based on morphology, as in [25,41,42,70]. However, it is important to state that, even with limited sampling, molecular analyses do not clearly support important subdivisions of the Symphypleona. For instance, Schneider et al. [65] tree does not support the Dicyrtomidae as a family, Yu et al. [66] do not support the Sminthurididae, and Yu et al. [66], Sun et al. [68] and Cucini et al. [69] do not support the suborder Appendiciphora. These are all well-delimited taxa based on morphology [26], and poor and uneven sample size and choice of markers could be blamed for such results. Nevertheless, more detailed phylogenetic analyses of the Symphypleona using modern tools are lacking, making its internal organization sometimes dubious and weakly supported in the light of the discovery of new taxa, as in the case of the Sminthuroidea. We hope in a near future more data on the systematics of the Symphypleona, as well as the Collembola as a whole, emerge to clarify its organization and to test different hypotheses concerning their evolution.

## 5. Conclusions

In this paper we described the highly troglomorphic *Troglobentosminthurus* gen. nov. from a cave system in the Brazilian semiarid region. The new genus shows a remarkable morphology, with troglomorphisms not recorded before in any Brazilian species of springtails and intermediate traits which made us revise the diagnosis of the Sminthurinae and discuss the exclusive morphology of the Bourletiellidae. *Troglobentosminthurus luridus* gen. nov. sp. nov. is discovered at the same time the Brazilian laws regarding cave protection become more flexible to favor commercial expansion, while the cave system where the species inhabits is developed in a karst already threatened by deforestation. Urgent conservation strategies are clearly in need to preserve this new taxon.

## Figures and Tables

**Table 1:** Comparison between the subfamilies of Sminthuridae.

Subfamilies/ Features	Antennae/Body Ratio	Ant. IV Subsegments	Distal Whorl of Chaetae on Tibiotarsi	Pretarsal Chaetae	Ungues with Cavity and/or a Filament-Like Tunica	Neosminthuroid Chaetae on the Parafurcal Area	Males’ Abd. VI Ventrally with a Pair of Spines	Dens Ventral Chaetae	Mucronal Apex
Sminthurinae	=/(>)	9–46	8–(9)	1–(2)	−	+/(−)	−	9–15	(sym)/asym
Songhaicinae	variable	10–26	6 or less	1–(2)	+	+/(−)	−	5–13	sym
Sphyrothecinae	<	0–12 *	7–9	2	−	+	+	0–6	asym

Data based in: [4,5,25,26,27,29,45,46,47,48,49,50] and the new genus herein described. Only extant genera were considered. Legends: ‘+’ present, ‘−’ absent, ‘=’ subequal; ‘>’ longer than; ‘<’ shorter than; ‘sym’ symmetrical; ‘asym’; asymmetrical; ‘( )’ more commonly seen; ‘*’ subsegments on the Ant. IV of the Sphyrothecinae may be somewhat vestigial.

**Table 2:** Comparison between the genera of the *Temeritas*-group (updated from [29]).

Genera/ Features	Ant. IV Subsegments	Eyes	Head Sexual Dimorphism	Antennae Sexual Dimorphism	Head Frontal Area Spines	Abdominal Dorso-Anterior Spines	Abdominal Dorso-Posterior Spines	Large Abdomen Sexual Dimorphism	Neosminthuroid Chaetae on Large Abdomen	Tibiotarsi distal Whorl Chaetae	Pretarsi Posterior Chaeta	Capitate Tenent-Hairs on Tibiotarsi II–III	Ungues Tunica	Ungues Pseudonychia	Ungues Inner teeth	Unguiculi Apical filament	Unguiculi Inner Teeth	Dens Ventral Chaetae	Mucronal Chaeta	Mucro Sexual Dimorphism
*Galeriella*	32	0 + 0	−?	−	−?	−?	−?	−?	−?	?	?	−	−	−	−	−	−	?	−	−?
*Keratosminthurus*	18–20	8 + 8	+	+	−	−	−	−	+	9	+	−	+/−	-	1	+	+/−	12–13	−	−
*Richardsitas*	28–30	8 + 8	−	−	+	+	+	+	−	9	+	+	−	+	1–2	+	+	13	+/−	−
*Temeritas*	18–46	8 + 8	−	−	+	+/−	−	+/−	−	9	+	−	+/−	+/−	1–2	+	+/−	13	+/−	−
*Troglobentosminthurus* gen. nov.	44	5 + 5	−	−	−	−	−	+	−	8–9	−	−	−	+	3–4	−	+	9	−	+

Data based in: [4,25,29,52,53,54,58] and the new genus herein described. Legends: ‘+’ present, ‘−’ absent, ‘?’ unknown, unclear.

## Data Availability

All data is contained within the article. All biological material is deposited at ISLA (# 79662 and 79664) as previously stated.
